# Cognitive Enhancers in Schizophrenia: A Systematic Review and Meta-Analysis of Alpha-7 Nicotinic Acetylcholine Receptor Agonists for Cognitive Deficits and Negative Symptoms

**DOI:** 10.3389/fpsyt.2021.631589

**Published:** 2021-04-06

**Authors:** María Recio-Barbero, Rafael Segarra, Arantzazu Zabala, Eduardo González-Fraile, Ana González-Pinto, Javier Ballesteros

**Affiliations:** ^1^Biocruces Bizkaia Health Research Institute, Barakaldo, Spain; ^2^Department of Psychiatry, Cruces University Hospital, Barakaldo, Spain; ^3^Department of Neurosciences, University of the Basque Country (UPV/EHU), Leioa, Spain; ^4^Centro de Investigación Biomédica en Red de Salud Mental, CIBERSAM, Madrid, Spain; ^5^Department of Health Sciences, International University of La Rioja, Logroño, Spain; ^6^Department of Psychiatry, Araba University Hospital, Vitoria-Gasteiz, Spain; ^7^Bioaraba Health Research Institute, Vitoria-Gasteiz, Spain

**Keywords:** nicotinic agonists, alpha-7 agonists, cognitive dysfunction, negative symptoms, schizophrenia

## Abstract

**Background:** Schizophrenia is a severe and enduring disease and is one of the leading causes of disability worldwide. Cognitive impairment is a core clinical symptom that plays a crucial role in functional outcomes and prognosis, thus making it a relevant treatment target. The aim of this study was to assess the efficacy of alpha-7 nicotinic acetylcholine receptor agonists (α7 nAChR) as adjunctive treatment to enhance cognition and ameliorate negative symptoms in patients with schizophrenia.

**Methods:** A search strategy was developed for MEDLINE, Embase, and the Cochrane Central Register of Controlled Trials up to May 2019. We included randomized controlled trials (RCTs) that compared antipsychotic treatment plus α7 nAChR agonists with antipsychotic treatment plus placebo and determined their effects on the main cognitive domains proposed by the MATRICS initiative and on negative symptoms. Two authors independently reviewed study eligibility and data extraction and assessed the risk of bias of the studies included. According to the Grading of Recommendations Assessment, Development, and Evaluation (GRADE) framework, we used a random-effects model and assessed the quality of the evidence.

**Results:** Thirteen studies were included in the quantitative analysis. No differences were found in any of the cognitive domains assessed in four RCTs (*n* = 414). In contrast, nine RCTs (*n* = 978) presented a small effect in support of α7 nAChR agonists for negative symptoms [standardized mean difference −0.28, 95% CI (−0.56 to −0.00); *P* = 0.05], even though the confidence to support this evidence is low according to the GRADE system.

**Conclusions:** Current evidence is too weak to consider α7 nAChR agonists as an effective add-on treatment to antipsychotics to enhance cognition and negative symptoms.

## Introduction

Schizophrenia is a severe and enduring mental disorder and a worldwide health priority. It is the most prevalent psychotic disorder, with a global average incidence of 15.2 per 100,000 inhabitants and a male:female ratio of 1.4:1 (1). Schizophrenia is by far one of the leading causes of disability worldwide, generating a substantial economic burden, with an annual estimated cost ranging from US$94 million to US$102 billion (2). Clinically, schizophrenia is characterized by a heterogeneous range of symptoms classified under the following domains: positive symptoms (e.g., delusions and hallucinations), negative symptoms (e.g., affective flattening, alogia, apathy, and social isolation), cognitive deficits (e.g., speed of processing and attention deficits), and affective symptoms (e.g., hypothymia, mania, and anxiety) (3).

Most antipsychotic agents have demonstrated efficacy in improving positive symptoms; however, data on their efficacy in negative symptoms and cognitive deficits are limited (4–6). Cognitive deficits are considered one of the most disabling symptoms and are considered one of the best predictors of long-term functional disability (7–10). Such recognition has been the main promoter for the development of new therapeutic approaches under the premise that improving cognitive impairments can indirectly result in improvements in patients' psychosocial functioning (11, 12). As a consequence, cognition has become a priority treatment target.

The two main approaches that have been developed are the psychosocial approach, which is based mainly on cognitive rehabilitation programs, and the pharmacological approach, which is based on the development of cognitive enhancers evaluated under the Measurement and Treatment Research to Improve Cognition in Schizophrenia (MATRICS) initiative.

Among the potential pharmacological therapeutic targets for developing cognitive enhancers, the MATRICS initiative has stressed the importance of the cholinergic system (13). This pathway has been widely associated with the modulation of cognitive processes (e.g., attention and memory) in healthy people. Donepezil and galantamine, which delay cognitive impairment in the earlier stages of Alzheimer's disease by acting on the cholinergic system (14), were among the first cognitive enhancers studied in schizophrenia. Nevertheless, a recent meta-analysis analyzing the efficacy of acetylcholinesterase inhibitors for cognitive deficits in schizophrenia has shown inconclusive results (15).

During the last decade, nicotinic acetylcholine receptors (nAChR) have been identified as a promising new therapeutic target. Specifically, the nicotinic subtype alpha-7 has been proposed as a potential target for the pharmacological treatment of neurocognitive impairment (16). Although evidence on the efficacy of nicotinic acetylcholine agonists is based on preclinical research (17), some preliminary clinical evidence has shown promising results. Postmortem studies have revealed reduced availability of α7 binding receptors (17) and reduced α7 expression in specific brain regions in schizophrenia, e.g., the hippocampus and frontal cortex (18). Studies addressing the presence of impairments in sensory gating report a decreased ability to gate auditory and eye-tracking stimuli, primarily due to alteration of chromosome 15 (18). Given that this chromosome contains the *CHRNA7* gene, which encodes the α7 nAChR (18), such results seem to suggest the presence of an impaired cholinergic neurotransmission signal (19, 20). Various clinical studies have described the positive influence of nicotine in diverse cognitive domains of patients with schizophrenia, such as attention and working memory processes (21–23). Hence, pharmacological compounds targeting nicotinic receptors have been considered potentially useful in the treatment of these deficits. More specifically, pharmacological treatment targeting the α7 nAChR is as a promising option that can enhance cognitive processes and ameliorate negative symptoms (24).

Recent trials have tried to prove the procognitive effect of new compounds targeting the α7 nAChR as add-on treatment to antipsychotic regimens, and in recent years, the several compounds identified as α7 nAChR agonists include RG3487, TC-5619, ABT-126, DMXB-A, and tropisetron. Despite the promising results for α7 nAChR agonists, evidence for their efficacy as procognitive drugs remains inconclusive (25). This study aims to summarize evidence on the efficacy of α7 nAChR agonists vs. placebo when used as adjunctive treatment to ameliorate cognitive and negative symptoms in patients with schizophrenia.

## Methods

### Type of Studies

Articles were included if they fulfilled the following conditions:

- Inclusion of patients diagnosed with schizophrenia or schizophreniform, schizoaffective, delusional, or psychotic disorder not otherwise specified according to the diagnostic criteria of the Diagnostic and Statistical Manual of Mental Disorders (DSM) (DSM-III-R, DSM-IV-R, or DSM-V) (3) or the International Classification of Mental Diseases (ICD) (ICD-9 or ICD-10) (26).
- Randomized placebo-controlled or double-blind clinical trials, open-label studies, or crossover studies. We excluded case series, observational designs, and studies with an *n* = 1 design. We compared cognitive enhancers as add-on treatments with add-on placebo to detect possible effects. If an effect existed and was clinically relevant, we extended the comparison to include several cognitive enhancers as competitive treatments.

No language restrictions were imposed in the choice of articles.

### Participants and Interventions

We included trials with patients diagnosed with schizophrenia or a schizophreniform, schizoaffective, delusional, or psychotic disorder not otherwise specified, according to DSM (3) or ICD (26) criteria. Patients had to be clinically stable and treated with typical or atypical antipsychotic agents. Experimental interventions were α7 nAChR as adjunctive medication to antipsychotic treatment. We included α7 nAChR agonists irrespective of the duration of treatment and the doses. The comparator intervention was matching placebo as adjunctive medication to antipsychotic treatment.

### Outcomes

#### Primary Outcome—Cognitive Domains

We assessed efficacy according to the results of cognitive tests and batteries based on the main cognitive domains affected in schizophrenia proposed by the MATRICS initiative: attention/vigilance, speed of processing, working memory, verbal learning, visual learning, problem solving, and social cognition (7). For each domain, we selected those psychometric measures that were replicated across trials or considered representative of a specific domain. For this purpose, we used the systematic review of Bakkour et al. (27) and the monograph of Lezak et al. (28) as general guidelines to identify and categorize the main cognitive domains evaluated through each cognitive test. Table 1 illustrates the main cognitive tests and batteries included clustered under the main domains proposed by the MATRICS initiative (7).

**Table 1:** Characteristics of the studies included in the systematic review and meta-analysis of cognitive deficits and negative symptoms in schizophrenia.

**Trial name**	**RCT design follow-up**	**Population**	**Interventions**	**Cognitive domains**	**Negative symptoms**
Freedman et al. (29)	Crossover 4 weeks	Outpatients; 9 women, 22 men; age range 22–60. DSM-IV-TR diagnosis of schizophrenia with stable symptoms, treated with a stable dose of typical or atypical antipsychotics. *Exclusion criteria*: substance abuse, smoking in the previous month, and women of fertile age	*DMXB-A* (75 or 150 mg/day) plus typical or atypical antipsychotic treatment vs. *placebo* plus typical or atypical antipsychotic treatment	MATRICS (MCCB) *Speed of processing*: BACS symbol coding, animal naming, TMT-A *Attention*: CPT-IP *Working memory*: WMS-III: spatial span and letter–number span *Verbal learning*: HVLT-R *Visual learning*: BVMT-R *Reasoning/problem solving*: NAB: Mazes *Social cognition*: MSCEIT	Scale for the Assessment of Negative Symptoms (SANS)
Haig et al. (30)	Parallel 12 weeks	Outpatients; 72 women, 131 men; age range 22–60. DSM-IV-TR diagnosis of schizophrenia confirmed by the 6.0 Mini International Neuropsychiatric Interview with stable symptoms, treated with a stable dose of an atypical antipsychotic. *Exclusion criteria*: substance abuse, treatment with clozapine, tricyclic antidepressants, or monoamine oxidases (MAOs)	*ABT-126* (10 or 25 mg/day) plus atypical antipsychotic vs. *placebo* plus atypical antipsychotic treatment	MATRICS (MCCB) *Speed of processing*: BACS symbol coding, animal naming, TMT-A *Attention*: CPT-IP *Working memory*: WMS-III: spatial span and letter–number span *Verbal learning*: HVLT-R *Visual learning*: BVMT-R *Reasoning/problem solving*: NAB: Mazes *Social cognition*: MSCEIT	Negative Symptom Assessment (NSA-16)
Haig et al. (31)	Parallel 12 weeks	Outpatients; 171 women, 190 men; age range 20–65. DSM-IV-TR diagnosis of schizophrenia with stable symptoms, treated with a stable dose of atypical antipsychotics. *Exclusion criteria*: substance abuse, treatment with mood stabilizers, tricyclic antidepressants, MAOs, and patients with doses >100 mg/day of clozapine	*ABT-126* (25, 50, or 75 mg/day) plus typical or atypical antipsychotic treatment vs. *placebo* plus typical or atypical antipsychotic treatment	MATRICS (MCCB) *Speed of processing*: BACS symbol coding, animal naming, TMT-A *Attention*: CPT-IP *Working memory*: WMS-III: spatial span and letter–number span *Verbal learning*: HVLT-R *Visual learning*: BVMT-R *Reasoning/problem solving*: NAB: Mazes *Social cognition*: MSCEIT	Negative Symptom Assessment (NSA-16)
Haig et al. (32)	Parallel 12 weeks	Outpatients; 32 women, 121 men; age range 22–60. DSM-IV-TR diagnosis of schizophrenia confirmed by the 6.0 Mini International Neuropsychiatric Interview with stable symptoms, treated with a stable dose of atypical antipsychotics. *Exclusion criteria*: substance abuse, treatment with mood stabilizers, tricyclic antidepressants, MAOs, and patients with doses >100 mg/day of clozapine	*ABT-126* (25 or 75 mg/day) plus atypical antipsychotic treatment vs. *placebo* plus antipsychotic treatment	MATRICS (MCCB) *Speed of processing*: BACS symbol coding, animal naming, TMT-A *Attention*: CPT-IP *Working memory*: WMS-III: spatial span and letter–number span *Verbal learning*: HVLT-R *Visual learning*: BVMT-R *Reasoning/problem solving*: NAB: Mazes *Social cognition*: MSCEIT	Negative Symptom Assessment (NSA-16)
Keefe et al. (33)	Parallel 12 weeks	Outpatients; 102 women, 215 men; age range 20–55. DSM-IV-TR diagnosis of schizophrenia or schizoaffective disorder with stable symptoms, treated with	*Encenicline* (0.27 or 0.9 mg/day) plus atypical antipsychotic treatment vs. *placebo* plus atypical antipsychotic treatment	MATRICS (MCCB) *Speed of processing*: BACS symbol coding, animal naming, TMT-A *Attention*: CPT-IP *Working memory*: WMS-III: spatial span and letter–number span	Positive and Negative Syndrome Scale (PANSS)
		a stable dose of atypical antipsychotics. *Exclusion criteria*: treatment with clozapine, antipsychotic polytherapy, and treatment with antidepressants (except for SSRIs)		*Verbal learning*: HVLT-R *Visual learning*: BVMT-R *Reasoning/problem solving*: NAB: Mazes *Social cognition*: MSCEIT	
Kem et al. (34)	Parallel 4 weeks	Outpatients; 20 women, 60 men; age range 18–60. DSM-IV diagnosis of schizophrenia with stable symptoms, treated with a stable dose of typical or atypical antipsychotics. *Exclusion criteria*: substance abuse and treatment with clozapine	*DMXB-A-ER* 150 mg/day plus typical or atypical antipsychotic drugs vs. *placebo* plus typical or atypical antipsychotic drugs	MATRICS (MCCB) *Speed of processing*: BACS symbol coding, animal naming, TMT-A *Attention*: CPT-IP *Working memory*: WMS-III: Spatial span and letter–number span *Verbal learning*: HVLT-R *Visual learning*: BVMT-R *Reasoning/problem solving*: NAB: Mazes *Social cognition*: MSCEIT	Scale for the Assessment of Negative Symptoms (SANS)
Lieberman et al. (35)	Parallel 12 weeks	Outpatients; 57 women, 128 men; age range 16–60. DSM-IV diagnosis of schizophrenia with stable symptoms, treated with a stable dose of quetiapine or risperidone	*TC-5619* (in increasing doses up to 25 mg/day) plus quetiapine or risperidone vs. *placebo* plus quetiapine or risperidone	CogState Battery *Speed of processing*: detection test *Attention*: identification test *Working memory*: one-back test and two-back test *Verbal learning*: International Shopping List Test *Visual learning*: Continuous Paired Associate Learning Test and One Card Learning Test *Reasoning/problem solving*: Groton Maze Learning Test and set-shifting test *Social cognition*: social–emotional test	Positive and Negative Syndrome Scale (PANSS)
Olincy et al. (36)	Parallel 1 day	Outpatients; 4 women, 8 men; age range 20–58. DSM-IV diagnosis of schizophrenia with stable symptoms, treated with a stable dose of typical or atypical antipsychotics	*DMXB-A* (75 mg/day plus 37.5 mg/day or 150 mg/day plus 75 mg/day) plus typical or atypical antipsychotic drugs vs. *placebo* plus typical or atypical antipsychotic drugs	RBANS *Attention*: digit span and coding *Immediate memory*: list learning and memory story *Delayed memory*: list recall, list recognition, story recall, and figure recall *Visuospatial/construction*: figure copy and line orientation *Language*: picture naming and semantic fluency	Not assessed
Preskorn et al. (37)	Parallel 21 days	Outpatients; 6 women, 15 men; age range 18–55. DSM-IV diagnosis of schizophrenia with stable symptoms, treated with a stable dose of aripiprazole, olanzapine, paliperidone, or risperidone	*EVP-6124* (0.3 or 1.0 mg/day) plus aripiprazole, olanzapine, paliperidone, or risperidone vs. *placebo* plus aripiprazole, olanzapine, paliperidone, or risperidone	CogState Battery *Speed of processing*: detection test *Attention*: identification test *Working memory*: one-back test and two-back test *Verbal learning*: International Shopping List Test *Visual learning*: Continuous Paired Associate Learning Test and One Card Learning Test *Reasoning/problem solving*: Groton Maze Learning Test and set-shifting test *Social cognition*: social–emotional test	Positive and Negative Syndrome Scale (PANSS)
Shinna et al. (38)	Parallel 8 weeks	Outpatients; 21 women, 19 men; age range 21–48. DSM-IV-TR diagnosis of schizophrenia with stable symptoms, treated with a stable dose of risperidone (2–6 mg/day). *Exclusion criteria*: substance abuse	*Tropisetron* 10 mg/day plus risperidone (2 to 6 mg/day) vs. *placebo* plus risperidone (2 to 6 mg/day)	CANTAB *Speed of processing*: simple and 5-choice (RTI) *Attention*: rapid visual information processing (RVP) *Working memory*: spatial working memory (SWM) *Verbal learning*: verbal recognition-recall memory (VRM) *Visual learning*: Paired Associate Learning (PAL) *Reasoning/problem solving*: Intra/Extradimensional Set Shifting and One Touch Stockings of Cambridge (OTS) *Social cognition*: Emotion Recognition Task (ERT)	Positive and Negative Syndrome Scale (PANSS)
Umbritch et al. (39)	Parallel 8 weeks	Outpatients; 66 women, 149 men; age range 15–55. DSM-IV-TR diagnosis of schizophrenia with stable symptoms, treated with a stable dose of risperidone (2–6 mg/day). *Exclusion criteria*: substance abuse	*RG3487* (5, 10, or 15 mg/day) plus risperidone (2–6 mg/day) vs. *placebo* plus risperidone (2–6 mg/day)	MATRICS (MCCB) *Speed of processing*: BACS symbol coding, animal naming, TMT-A *Attention*: CPT-IP *Working memory*: WMS-III: Spatial span and letter–number span *Verbal learning*: HVLT-R *Visual learning*: BVMT-R *Reasoning/problem solving*: NAB: Mazes *Social cognition*: MSCEIT	Positive and Negative Syndrome Scale (PANSS)
Walling et al. (40)	Parallel 24 weeks	Outpatients; 180 women, 297 men. DSM-IV-TR diagnosis of schizophrenia with stable symptoms treated with a stable dose of atypical antipsychotics. *Exclusion criteria*: substance abuse, treatment with clozapine, sertindole, melperone, mood stabilizers, antidepressants, or anxiolytics	*TC-5619* (5 or 50 mg/day) plus atypical antipsychotic vs. *placebo* plus atypical antipsychotic treatment	CogState Battery *Speed of processing*: detection test *Attention*: identification test *Working memory*: one-back test and two-back test *Verbal learning*: International Shopping List Test *Visual learning*: Continuous Paired Associate Learning Test and One Card Learning Test *Reasoning/problem solving*: Groton Maze Learning Test and set-shifting test	Positive and Negative Syndrome Scale (PANSS)
Zhang et al. (41)	Parallel 10 days	Inpatients; 10 women, 30 men; age range 20–55. DSM-IV-TR diagnosis of schizophrenia with stable symptoms, treated with a stable dose of risperidone (3–6 mg/day). *Exclusion criteria*: substance abuse and presence of a comorbid mood or anxiety disorder	*Tropisetron* (5, 10, or 20 mg/day) plus risperidone (3–6 mg/day) vs. *placebo* plus risperidone (3–6 mg/day)	RBANS *Attention*: digit span and coding *Immediate memory*: list learning and memory story *Delayed memory*: list recall, list recognition, story recall, and figure recall *Visuospatial/construction*: figure copy and line orientation *Language*: picture naming and semantic fluency	Not assessed

*MCCB, MATRICS Consensus Cognitive Battery; BACS, Brief Assessment of Cognition in Schizophrenia; TMT-A, Trail Making Test Part A; CPT-IP, The Continuous Performance Test-Identical Pairs version; WMS-III, Wechsler Memory Scale–Third Edition; HVLT-R, Hopkins Verbal Learning Test Revised; BVMT-R, Brief Visuospatial Memory Test-Revised; NAB, Neuropsychological Assessment Battery; MSCEIT, The Mayer–Salovey–Caruso Emotional Intelligence Test*.

#### Secondary Outcome—Negative Symptoms

For the assessment of negative symptoms, we considered those studies that used standardized instruments to evaluate negative clinical symptoms such as the Negative Symptoms Assessment Scale (NSA-16) (42), the Positive and Negative Syndrome Scale for Schizophrenia (PANSS) (43), and the Scale for Negative Symptoms Assessment (SANS) (Table 1) (44).

### Search Methods for the Identification of Studies

A search strategy was developed to identify potential studies (see search strategy in Supplementary Appendix 1). We developed a search strategy for MEDLINE (Ovid), Embase (Ovid), and the Cochrane Central Register of Controlled Trials (CENTRAL) and searched for RCTs up to May 2019. We performed the search without language or date restrictions. The references of potentially eligible trials and relevant reviews were searched for additional citations.

### Data Extraction and Analysis

We followed the Preferred Reporting Items for Systematic Reviews and Meta-Analyses (PRISMA) statement to design the search flowchart. After removing duplicate records, two independent and blinded review authors (JB, MR) screened the titles and abstracts of the studies obtained from the literature searches to assess eligibility. We obtained full reports from the studies considered to be potentially eligible. Disagreements were resolved by discussion and consensus.

The data extracted included the following: primary and secondary outcomes, number of patients included, population characteristics (including age, sex, duration of illness, and diagnosis), smoking status, duration of treatment, outcome scales used, and tests for cognitive and negative assessment (i.e., mean, standard deviation). In case of missing outcome data, the corresponding author of the study was contacted via email.

The study characteristics and quantitative data were extracted using a data extraction sheet form. Sample sizes, means, and standard deviations from each intervention were obtained for each relevant outcome to calculate standardized mean differences (SMDs). We applied SMDs owing to the different measures, clinical tests, and metrics used to assess cognitive deficits and negative symptoms. We interpreted SMDs as small effect, medium effect, or large effect according to Cohen's criteria, with 0.2 representing a small effect, 0.5 a moderate effect, and 0.8 a large effect (45).

After a training exercise, two independent authors (JB, MR) assessed the risk of bias for each trial included to determine study quality according to the PRISMA guidelines and the Cochrane Handbook of Systematic Reviews of Interventions (46). Risk of bias was classified as low, unclear, or high. Disagreements were resolved by discussion and consensus. We used a random-effects model to combine individual effect sizes (47).

We used the *I*^2^ statistic to assess the heterogeneity of the studies. The *I*^2^ statistic quantifies inconsistencies between the studies. We considered *I*^2^-values >60% as indicating substantial heterogeneity and conducted a subgroup analysis if feasible to explain possible sources of heterogeneity. All analyses were performed using R (https://cran.r-project-org/), specifically the packages “meta” and “metaphor” (48, 49).

Finally, we assessed reporting bias depending on the availability of the study protocols described in the methodology section of each trial. The overall quality of evidence for each cognitive and negative measure was presented according to the Grading of Recommendations Assessment, Development, and Evaluation (GRADE) approach (50). We judged the quality of evidence considering the following dimensions: (a) study limitations (risk of bias across the studies at the outcome level), (b) inconsistency, (c) imprecision, (d) indirectness, and (e) publication bias. Two review authors (JB, MR) independently rated the quality of evidence for each outcome as high, moderate, low, or very low according to the GRADE framework (50). To generate an evidence table for each comparison, we used the online Guideline Development Tool (https://gradepro.org).

## Results

### Characteristics of the Studies Included and Patients

**Figure 1:**
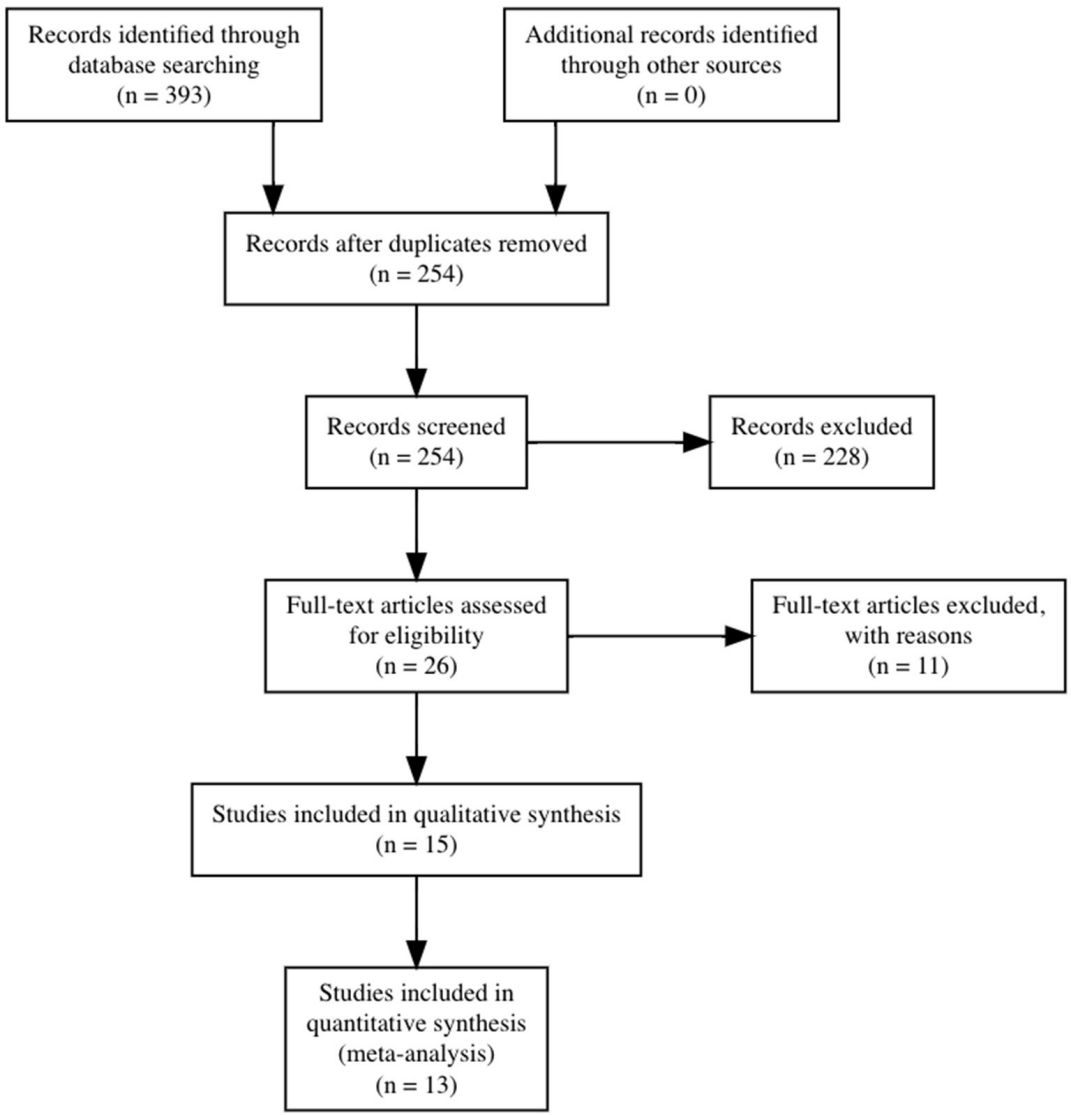
Preferred Reporting Items for Systematic Reviews and Meta-analysis (PRISMA) flow diagram.

Our search identified 393 articles. After excluding duplicates, 254 references were reviewed by title and abstract, and 15 studies were considered eligible and assessed as full text; of these, 13 studies were included in the present review. Four studies were included in the quantitative analysis for cognitive outcomes and nine studies were included in the quantitative analysis for negative symptoms (Figure 1).

Thirteen studies compared the effectiveness of antipsychotics plus α7 nAChR agonists with antipsychotics plus placebo for treatment of cognitive deficits. Of these, three studies used ABT-126 (30–32) or DMXB-A (29, 34, 36), two studies used TC-5619 (35, 40) or tropisetron (38, 41), and one study used encenicline (33), EVP-6124 (37), or RG 3487 (39).

To assess the effectiveness of antipsychotics plus α7 nAChR agonists vs. placebo in negative symptoms, we finally included for analysis three studies using ABT-126 (30–32), two studies using tropisetron (38, 51) or DMXB-A (29, 34), and one study using RG 3487 (39) or citicoline (52) (Table 1). The sample size of the studies ranged from 12 to 477 participants, with a median of 153 participants (IQR 40 to 215).

### Risk of Bias of the Studies Included in the Qualitative Synthesis

**Figure 2:**
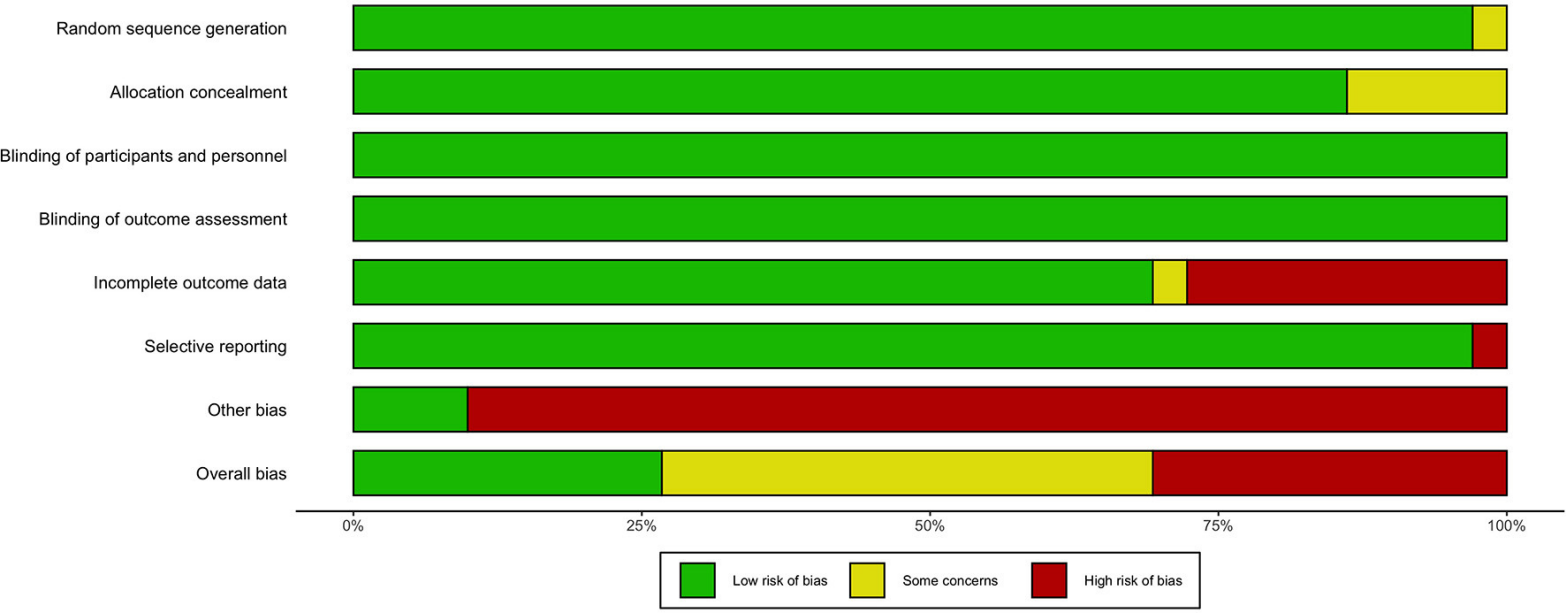
Risk of bias summary of included studies.

The overall risk of bias across the studies is presented in Figure 2. Except for two studies (29, 41), all studies were reported as randomized and provided appropriate information to assess the randomization sequence procedure. Overall, the risk of bias of random sequence generation was rated as low. Five studies (29, 33, 39, 41, 52) presented an unclear risk for allocation concealment. Overall, the risk of bias of allocation concealment was rated as unclear. All studies reported appropriate information for blinding conditions regarding participants and personnel and outcome assessment. The risk of bias for blinding participants, personnel, and assessors was rated as low for all outcomes. One study presented an unclear risk for incomplete data outcomes (29), since it did not provide data on loss or withdrawal of patients. The study by Haig et al. (31) was rated as having a high risk of bias, since when losses in treatment were described, differences were observed between the treatment arms or between the interventions. Overall, the risk of bias for incomplete outcome data was rated low for all outcomes. As for selective reporting, all the studies reported on clinical trial registration. However, one study (33) was reported as having an unclear risk for selective reporting due to primary and secondary outcome-specific variables that were not provided in the clinical trial protocol. We also considered that Freedman et al. (29) presented a high risk of bias because the variables and measures reported in the registered clinical trial protocol did not coincide with those presented in full-text articles. Finally, except for the studies carried out by Zhang et al. (41) and Nozoorian et al. (51), all the other studies included in the qualitative analysis presented a strong suspicion of publication bias owing to the direct participation of the pharmaceutical industry.

### Effects of Interventions

**Figure 3:**
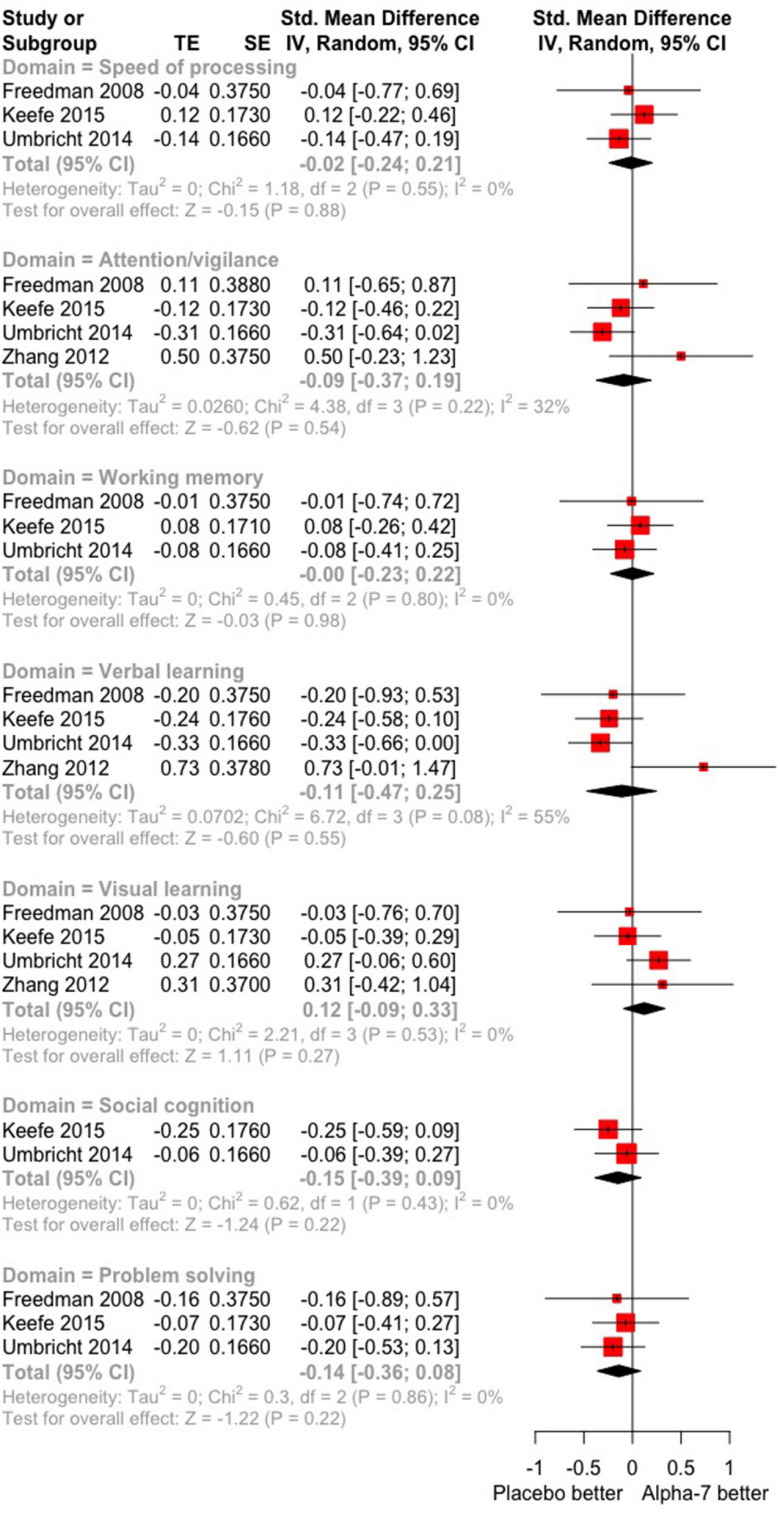
Overall efficacy by cognitive domain of antipsychotic drug plus α-7 nAChR agonists vs. antipsychotic drug plus placebo.

A summary of the main findings is presented in Figure 3.

#### Primary Outcome—Cognitive Deficits

##### Speed of Processing

Based on all available evidence (three studies, including 376 randomized participants: 268 on α7 nAChR agonists, 108 on placebo), and given the low quality of evidence, adding α7 agonists to antipsychotic treatment has little or no effect on speed of processing. SMDs are compatible with small to moderate effects, supporting either α7 agonists or placebo (SMD −0.02, 95% CI −0.24 to 0.20; *P* = 0.86) (Figure 3). Effect estimates were not statistically heterogeneous (χ^2^ = 1.22 on 2 *df*, *P* = 0.54, *I*^2^ = 0%). We downgraded the quality of evidence owing to a strong suspicion of publication bias.

##### Attention

Based on three studies including 413 randomized participants (296 on α7 nAChR agonists, 117 on placebo) and providing low-quality evidence, adding α7 agonists to antipsychotic treatment may have little or no effect on attention. SMD values showed a compatible small to moderate effect supporting either α7 agonists or placebo (SMD −0.09, 95% CI −0.37 to 0.02, *P* = 0.56) (Figure 3). Heterogeneity in effect estimates was low (χ^2^ = 4.51 on 3 *df*, *P* = 0.21, *I*^2^ = 34%). We downgraded the quality of evidence owing to a strong suspicion of publication bias.

##### Working Memory

Based on three studies, including 380 randomized participants (268 on α7 nAChR agonists, 112 on placebo) and providing low-quality evidence, adding α7 agonists to antipsychotic treatment may have little or no effect on working memory. The SMD showed a small to moderate effect supporting either α7 agonists or placebo (SMD 0.00, 95% CI −0.23 to 0.22, *P* = 0.97) (Figure 3). Effect estimates were not statistically heterogeneous (χ^2^ = 0.42 on 2 *df*, *P* = 0.81, *I*^2^ = 0%). We downgraded the quality of evidence owing to a strong suspicion of publication bias.

##### Verbal Learning

Based on four studies including 414 randomized participants (296 on α7 nAChR agonists, 118 on placebo) and providing low-quality of evidence, adding α7 agonists antipsychotic treatment had little or no effect on verbal learning. SMD values are compatible with a small to moderate effect supporting α7 agonists and moderate effects supporting placebo (SMD −0.11, 95% CI −0.47 to 0.25, *P* = 0.55) (Figure 3). Effect estimates were moderately heterogeneous (χ^2^ = 6.72 on 3 *df*, *P* = 0.08, *I*^2^ = 55%). We downgraded the quality of evidence owing to a strong suspicion of publication bias.

##### Visual Learning

Based on four studies, including 414 randomized participants (296 on α7 nAChR agonists, 118 on placebo) and providing low-quality evidence, adding α7 agonists to antipsychotic treatment may have little or no effect on visual learning. SMD values are compatible with a small to moderate effect supporting α7 agonists and a very small effect supporting placebo (SMD 0.12, 95% CI −0.09 to 0.34, *P* = 0.26) (Figure 3). Effect estimates were not statistically heterogeneous (χ^2^ = 2.22 on 3 *df*, *P* = 0.53, *I*^2^ = 0%). We downgraded the quality of evidence owing to a strong suspicion of publication bias.

##### Problem Solving

Based on three studies, including 376 randomized participants (268 on α7 nAChR agonists, 108 on placebo) and providing low-quality evidence, adding α7 agonists to antipsychotic treatment may have little or no effect on reasoning/problem solving. The SMD values are compatible with a very small effect supporting α7 agonists and a moderate effect supporting placebo (SMD −0.14, 95% CI −0.36 to 0.08, *P* = 0.22) (Figure 3). Effect estimates were not statistically heterogeneous (χ^2^ = 0.28 on 2 *df*, *P* = 0.87, *I*^2^ = 0%). We downgraded the quality of evidence owing to a strong suspicion of publication bias.

##### Social Cognition

Based on two studies including 345 randomized participants (248 on α7 nAChR agonists, 97 on placebo) and providing low-quality evidence, α7 agonists have little or no effect on problem solving. The SMD values are compatible with a minimal effect supporting α7 agonists and a moderate effect supporting placebo (SMD −0.15, 95% CI −0.39 to 0.08, *P* = 0.20) (Figure 3). Effect estimates were not statistically heterogeneous (χ^2^ = 0.62 on 1 *df*, *P* = 0.43, *I*^2^ = 0%). We downgraded the quality of evidence owing to a strong suspicion of publication bias.

#### Secondary Outcome—Negative Symptoms

**Figure 4:**
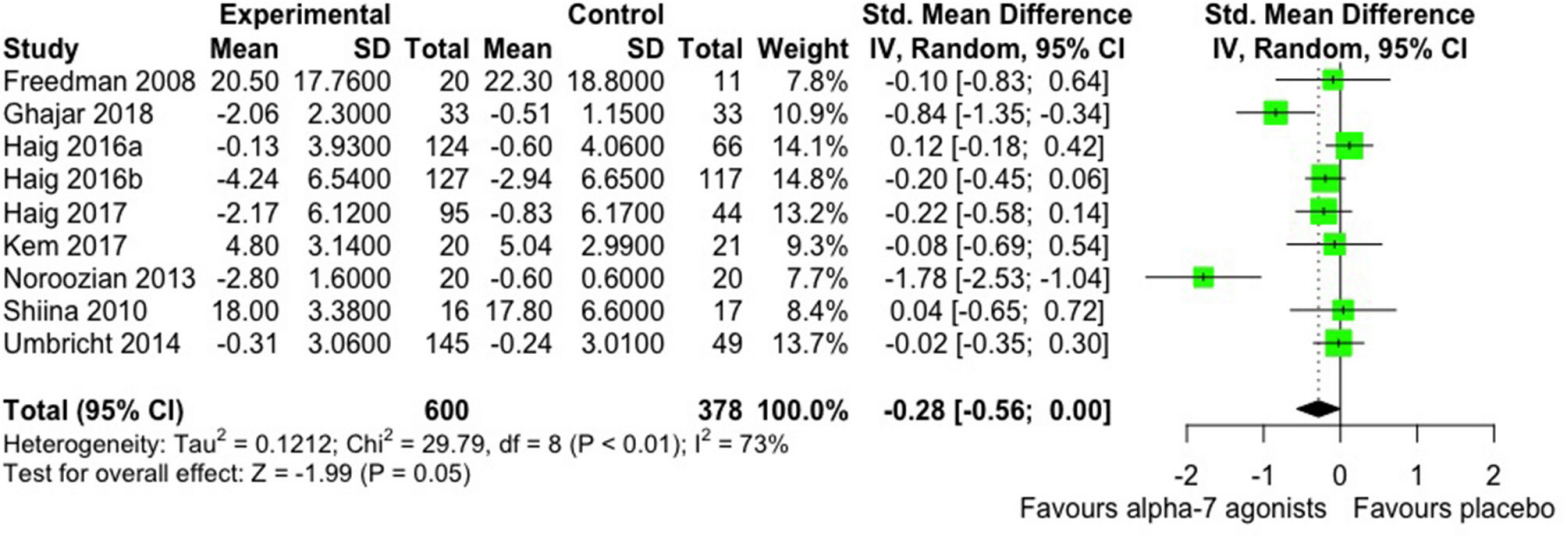
Overall efficacy in negative symptomatology of antipsychotic drug plus α-7 nAChR agonists vs. antipsychotic drug plus placebo.

Based on nine studies including 978 randomized participants (600 on α7 nAChR agonists, 378 on placebo) and providing low-quality evidence, adding α7 agonists to antipsychotic treatment may have a low to moderate effect on negative symptoms. The SMD values are compatible with a moderate effect supporting α7 agonists over placebo (SMD −0.28, 95% CI −0.56 to −0.00, *P* = 0.05) (Figure 4). Effect estimates were statistically heterogeneous (χ^2^ = 29.79 on 8 *df*, *P* < 0.001, *I*^2^ = 73%). Considering such heterogeneity, we observed that the study of Noroozian et al. (51) had a major impact on overall heterogeneity and effect size. Its removal from the evidence set decreased *I*^2^ from 73 to 55% and the SMD to −0.15 (95% CI −0.39, 0.08). Whereas, heterogeneity between effects across studies decreased, the SMD also decreased from −0.28 to −0.15. We downgraded the quality of evidence owing to a strong suspicion of publication bias and unexplained heterogeneity (inconsistency).

## Discussion

This systematic review and meta-analysis examined the efficacy of adding α7 nAChR agonists to antipsychotic treatment in cognitive deficits and negative symptoms in patients diagnosed with a schizophrenia spectrum disorder. Overall, α7 agonists may be more efficacious than placebo, even though the quality of evidence remains low owing to uncertainty over the quality of evidence.

Our main finding was that there was no general effect of α7 nAChR agonists as adjunctive treatment on cognitive deficits. Our results support the view that adding α7 nAChR agonists to habitual antipsychotic medication has little or no effect on any of the cognitive domains evaluated. We downgraded the quality of evidence to low quality in all of the cognitive domains assessed owing to suspected publication bias. Consistent with our results, a 2017 systematic review and meta-analysis (53) analyzing the effect of α7 and α4β2 receptor agonists in patients with schizophrenia did not reveal an overall improvement in cognitive (or negative) outcomes when measuring composite cognitive scores. Moreover, in this study, the individual cognitive domains typically affected in these patients were not taken into consideration separately.

We did not evaluate the effect of α7 nAChR agonists on overall cognitive function, as we do not consider that a composite neurocognitive measure is a valid representative measure for the evaluation of the real cognitive effects of a specific treatment. Thus, we avoided this problem by evaluating the main cognitive domains proposed by the MATRICS initiative (7) to estimate the effect of these treatments on cognitive functioning. Our view is that the assessment of individual cognitive domains is essential for estimating the actual effect of those treatments on the cognitive areas that are prototypically affected in schizophrenia. Besides, this approach is of particular importance in the social cognition domain, as the effect observed has been shown to be strongly correlated with the daily functioning of schizophrenia patients (54, 55).

There was no evidence of heterogeneity in any specific cognitive domain (*I*^2^ < 60%). As mentioned previously, evidence of reporting bias was suspected for all cognitive domains, owing to partial presentation of raw data for specific cognitive domains. Reporting of data in both full-text format and as supplemental material was deficient in most studies, except the four studies included in the quantitative analysis (29, 33, 39, 41).

Regarding negative symptoms, α7 nAChR agonists do present moderate effects as adjunctive treatment on negative symptoms. Although there was considerable heterogeneity among the studies reporting negative symptoms (*I*^2^ = 73%), this situation does not seem to be accounted for by other clinical moderators. For that reason, we did not perform a subgroup analysis. We did indeed influence analyses by removing one study at a time from the evidence set. As presented in the results section, the residual heterogeneity observed after removing the study of Noroozian et al. (51) could be explained using a random-effects analysis model, in which it is considered that the studies included are a random sample of the population of all studies and are weighted. Finally, we downgraded the quality of evidence because of a strong suspicion of publication bias with respect to negative symptoms.

Only four of 13 studies complied with the CONSORT guidelines for reporting clinical trials and presented raw data to assess the efficacy of α7 nAChR agonists on cognitive deficits. It is remarkable that even though cognition was considered the primary measure in most of the studies included, the completeness of information was greater for clinical outcomes, with a total of nine trials providing either full-text data or Supplementary Material for the assessment of negative symptoms. Transparency and completeness in reporting biomedical research are essential when evaluating the methodological quality and reproducibility of clinical trials. Despite the development of the CONSORT statement in 2010 to provide an evidence-based checklist of recommendations for reporting the findings of randomized clinical trials, a large proportion of biomedical trials do not yet provide sufficiently high-quality reporting.

Most of the clinical trials included in this review did not report a duration of treatment beyond 12 weeks, except for that of Walling et al. (40), where follow-up was longer than the average and data were analyzed at weeks 12 and 24. Moreover, and from a clinical point of view, it is interesting to consider the potential effect of these prognostic drugs on patients' day-to-day functionality. While evidence has shown that improvements in cognitive functioning correlate with actual patient functioning, recommendations from institutions, such as the FDA-NIMH, have highlighted the need to include variables that assess the impact on patient functionality as co-primary measures (56). Despite this, only six studies considered the inclusion of measures to assess patients' functional capacity.

It is also necessary to consider that patients included in most clinical trials may not be representative of daily clinical practice, as inclusion criteria are often very restrictive (e.g., chronically ill patients with stable symptoms, higher rates of adherence, or patients not presenting another psychiatric comorbidity, such as substance abuse or dependence). Implementation of such strategies in daily clinical practice could be limited. Overall, the quality of evidence is low, suggesting that prescribing α7 nAChR agonists as adjunctive therapy does not seem to improve the prototypical clinical deficits of schizophrenia, such as negative and cognitive symptoms.

### Limitations

Our work is subject to a series of limitations.

First, we could not include some studies because the authors did not provide enough data, either as full text or Supplementary Material. In addition, most studies did not present appropriate information in the methods section to assess the risk of bias in the study and outcomes. None of the authors we contacted to obtain supplementary information and data for cognitive and negative measures responded to our requests. For this reason, the quantitative analysis for cognitive outcomes includes a very small number of studies.

The large number of cognitive tasks and batteries widely used in the literature to evaluate cognitive deficits in patients with schizophrenia made assessment of the intervention effects of a specific treatment over the years challenging. Additionally, while studies frequently report combined or pooled cognitive measures as a standard general measure of real cognitive functioning, composite measures are difficult to understand and interpret. Given this heterogeneity, the MATRICS initiative emerged to provide unified reporting criteria for the main cognitive processes affected in schizophrenia (7). Even if the MATRICS initiative is subject to methodological and clinical criticisms, it has made it possible to frame cognitive constructs and select standardized cognitive measures to assess cognitive functions.

The average duration of clinical trials is short. In general terms, clinical trials evaluating the effectiveness of cognitive enhancers in schizophrenia are shorter than 12 weeks. As stated by Green et al. (54), the desirable effects on cognition, and even more so on the patient's functioning, can be expected over a period of 6 to 12 months. As a result, the studies included may not have been sufficiently long to provide a genuine assessment of the long-term efficacy and safety of cognitive enhancers (54).

Since a wide range of cognitive alterations are present prior to the onset of the disease and these follow a stable course (57, 58), it is necessary to consider the inclusion of patients with a first psychotic episode. The inclusion of this group could help us to understand and evaluate those treatments in more recently diagnosed patients and, with it, prevent resistance to pharmacological treatment.

### Conclusions

To summarize, we found no evidence of the effectiveness of α7 nAChR as add-on treatment for cognitive deficits in schizophrenia. Although there is a small effect supporting the use of α7 nAChR agonists for negative symptoms, the general quality of evidence is low. To provide a more accurate picture of the actual effects of such treatments, we need adequately powered clinical trials, as well as a preregistration and/or clear reporting of methods and outcomes in accessible protocols, in order to prevent both reporting bias and publication bias.

## Data Availability Statement

The original contributions presented in the study are included in the article/Supplementary Material, further inquiries can be directed to the corresponding author/s.

## Author Contributions

MR-B, RS, AZ, EG-F, AG-P, and JB contributed to the design, development, and final draft of the protocol. EG-F, MR-B, and JB conducted the systematic literature search. RS and MR-B made the trial selection and obtained full-text reports. RS and AZ extracted the main study characteristics. MR-B and JB extracted effect sizes from the studies included, assessed the risk of bias, and assessed the quality of evidence (GRADE). JB led the data analysis. AZ, RS, AG-P, and MR-B assisted with data interpretation. All review authors contributed to the final draft.

## Conflict of Interest

The authors declare that the research was conducted in the absence of any commercial or financial relationships that could be construed as a potential conflict of interest.
