# Supplementary material for: Cognitive Enhancers in Schizophrenia: A Systematic Review and Meta-Analysis of Alpha-7 Nicotinic Acetylcholine Receptor Agonists for Cognitive Deficits and Negative Symptoms
Source: Front Psychiatry. 2021 Apr 6;12:631589. doi: 10.3389/fpsyt.2021.631589 (PMC8055861; doi:10.3389/fpsyt.2021.631589)
Supplement: Supplementary file 1 [file Data_Sheet_1.docx]

Supplementary Material

# Supplementary Data – Search strategy

- **Cochrane Central Register of Controlled Trials (CENTRAL)**

# 1 - alpha7 or alpha-7 or α7 or α-7 or alpha next 7 or α next 7

# 2 - in Trials

- **Embase**

# 1 – (alpha 7 or alpha7).af.

# 2 - (clin$ adj2 trial).mp. or ((singl$ or doubl$ or trebl$ or tripl$) adj (blind$ or mask$)).mp. or (random$ adj5 (assign$ or allocat$)).mp. or randomi$.mp. or crossover.mp. or exp randomized-controlled-trial/ or exp double-blind-procedure/ or exp crossover-procedure/ or exp single-blind-procedure/ or exp randomization/

# 3 - (schizo$ or psychotic$ or psychosis or psychoses).mp. or ((chronic$ or severe$ or persistent$) adj (mental$ or psychological$) adj (disorder$ or ill$)).mp. or exp schizophrenia/ or exp psychosis/ or mental patient/ or (tardiv$ adj dyskine$).mp. or neuroleptic agent/ or (neuroleptic$ and (malignant adj2 syndrome)).mp or tardive dyskinesia/ or akathisia/ or exp neuroleptic malignant syndrome/ or (neuroleptic$ and movement and disorder$).mp. or parkinsoni$.mp. or parkinson's.mp.

# 4 - 3 not parkinson’s.ti.

# 5 - 2 and 4

# 6 - 1 and 5
